# Human milk cortisol is inversely associated with infant BMI and mediates the association between maternal plasma and infant salivary cortisol concentrations

**DOI:** 10.1038/s41366-025-01815-4

**Published:** 2025-05-31

**Authors:** Ana Luz Kruger, Agustina Malpeli, Marisa Sala, Carla Casado, Ignacio Mendez, Lucrecia Fotia, Andrea Tournier, María Victoria Fasano, María F. Andreoli

**Affiliations:** 1 Instituto de Desarrollo e Investigaciones Pediátricas (IDIP), HIAEP “Sor María Ludovica” de La Plata—Comisión de Investigaciones Científicas de la Provincia de Buenos Aires (CIC-PBA), La Plata, Buenos Aires, Argentina; 2 CONICET, La Plata, Buenos Aires, Argentina; 3 Laboratorio Central, HIAEP “Sor María Ludovica” de La Plata, La Plata, Buenos Aires, Argentina; 4 Centro de Matemática de La Plata (CMaLP), Facultad de Ciencias Exactas, Universidad Nacional La Plata (UNLP)—CIC-PBA, La Plata, Buenos Aires, Argentina; 5 Department of Surgical Sciences, Functional Pharmacology and Neuroscience, Uppsala University, Uppsala, Sweden

**Keywords:** Obesity, Fat metabolism

## Abstract

**Purpose:**

The pathways through which milk cortisol affects infant body weight and adiposity remain poorly understood.

**Aims:**

To assess the influence of maternal weight status on maternal cortisol concentrations and infant outcomes; to evaluate the relationship between maternal plasma and milk cortisol concentrations and infant salivary cortisol, body weight and adiposity during the first 3 months of life in a cohort of exclusively breastfed infants; to determine whether milk cortisol mediates these effects; and to explore the association between infant salivary cortisol and measures of body weight and adiposity.

**Methods:**

In this prospective observational study, we measured cortisol concentrations in plasma and milk samples from lactating women at 10 days (*n* = 68) and 3 months postpartum (*n* = 34), and in saliva samples from their 3-month-old infants (*n* = 34). Multiple linear regression and mediation analysis were conducted to determine the relationship between maternal characteristics and infant anthropometric measurements or salivary cortisol concentration and whether they were mediated by milk cortisol.

**Results:**

Plasma and milk cortisol concentrations were inversely associated with gestational weight gain and postpartum weight retention at 10 days postpartum. Maternal plasma and milk cortisol concentrations were directly with infant salivary cortisol concentration [Beta (95% CI): 0.05 (0.00, 0.09), *p* = 0.038; 0.95 (0.51, 1.39), *p* < 0.001], and inversely associated with infant BMI *z*-score [Beta (95% CI): −0.11 (−0.17, −0.04), *p* = 0.004; −1.04 (−1.69, −0.39), *p* = 0.003] at 3 months of lactation, the former mediated by milk cortisol (*p* = 0.039). Infant salivary cortisol was not associated with body weight and adiposity at 3 months of lactation.

**Conclusion:**

Our study shows that in exclusively breastfed infants, milk cortisol is inversely associated with BMI *z*-score and influences salivary cortisol at 3 months postpartum. Further research is warranted to explore the mechanisms involved and how these interactions evolve across different stages of lactation.

**Trial registration:**

This study was registered at clinicaltrials.gov as NCT05798676.

## Introduction

Human lactation involves complex hormonal and metabolic adaptations in mothers to meet the nutritional and developmental needs of their infants. Human milk is an exceptionally nutrient-rich biological fluid containing immune factors and bioactive components that can influence the infant’s physiology via various mechanisms. These components play a critical role in promoting infant growth and development [1–3]. Among exclusively breastfed infants, where human milk serves as the primary source of nourishment, even subtle variations in milk composition may impact growth trajectories and developmental outcomes.

Glucocorticoids have gained attention as key components of human milk in recent years, reflecting maternal Hypothalamic-Pituitary-Adrenal (HPA) axis activity. Milk cortisol and cortisone exhibit a diurnal rhythm, peaking in the morning and reaching the lowest concentration at night [4], and were initially studied for their potential influence on offspring behavioral phenotypes [5, 6]. As cortisol is implicated in diverse physiological processes, including the metabolism of glucose and fats [7], it plays an important role in fat mass gain. However, the specific impact of milk cortisol on infant body weight and adiposity remains incompletely understood. While some studies report no significant association between milk cortisol and infant growth or body composition [8, 9], others suggest a positive link with adiposity [10] or a negative association with BMI percentile gain during the first 2 years of life [11], hinting at a protective role of milk cortisol against excessive adiposity in later life. These mixed findings highlight the need for further research, utilizing controlled sample collection within the context of exclusive breastfeeding, to better understand the role of milk cortisol in influencing infant weight and adiposity. In addition to cortisol, circulating hormones such as leptin, ghrelin, and liver-enriched antimicrobial peptide 2 (LEAP2) are known to regulate metabolism and body weight in adults. Since the first months of life represent a critical period for growth and adiposity programming [12, 13], these hormones have also been investigated for their potential roles in influencing infant growth and fat accretion [12, 14, 15]. However, the potential role of cortisol in this context is less well understood. A recent review and meta-analysis of cortisol measures and adiposity in children yielded inconclusive results [16]. In infants, cortisol is typically assessed through saliva as a non-invasive proxy for HPA axis activity [17], yet its relationship with infant body weight and adiposity and a possible association with maternal cortisol levels has not been studied before.

Maternal factors, including weight status and hormonal milieu, are critical in shaping milk composition [18] and directly influencing infant health and growth. Maternal weight status during pregnancy and lactation has been consistently linked to milk hormone concentrations [19, 20] and to offspring risks for obesity and type 2 diabetes [21]. Also, pre- and postnatal exposure to maternal stress and elevated cortisol levels have been associated with infant negative affectivity (sadness, fear, distress, and falling reactivity) [6, 22], and also with crying, fussing, and negative facial expressions [23]. Although these associations are well-documented, the complex dynamics of the mother-milk-infant triad—particularly the pathways through which maternal characteristics affect infant outcomes via milk composition—remain poorly understood. Traditional statistical methods for examining these relationships often adjust for intermediate variables, which can yield inaccurate estimates of both direct and indirect effects. Counterfactual-based mediation analysis, a novel method employing resampling techniques, offers a more robust framework for identifying mediation effects. While this method has been applied in a limited number of studies exploring maternal predictors of milk composition and their associations with infant outcomes [24, 25], further research is warranted. Specifically, investigations should aim to determine whether milk cortisol mediates the influence of maternal characteristics on infant cortisol levels, body weight, and adiposity.

To address these knowledge gaps, our study assessed the influence of maternal weight status on maternal cortisol concentrations. Additionally, we aimed to evaluate the relationship between maternal plasma and milk cortisol concentrations with infant outcomes—specifically body weight, adiposity, and salivary cortisol concentrations—during the first 3 months of life in a cohort of exclusively breastfed infants. Finally, we explored the association between infant salivary cortisol and body weight and adiposity measures. We hypothesize that maternal weight status and cortisol concentrations influence infant body weight, adiposity, and salivary cortisol levels, with these effects mediated by milk cortisol. Furthermore, we propose that infant salivary cortisol may play a role in weight gain and fat accretion. By addressing these questions, this study aims to enhance our understanding of the complex biological interactions within the mother-milk-infant triad.

## Materials and methods

### Subjects and study design

**Fig. 1:**
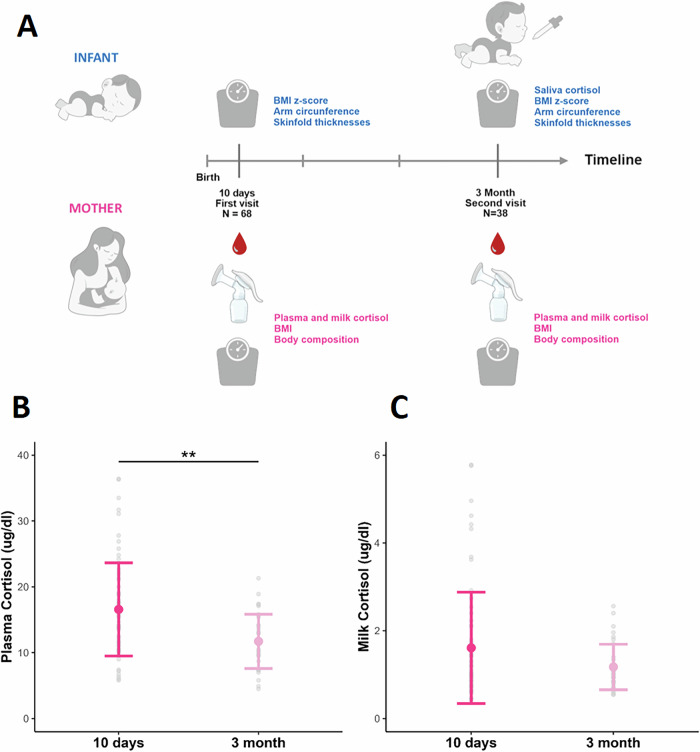
Experimental design. **A** Cortisol concentration in maternal plasma (**B**) and milk (**C**) at 10 days and 3 months postpartum. Concentrations are presented as geometric mean (95% CI). **p < 0.001 by Student’s test.

The study design of this prospective cohort observational study can be seen in Fig. 1A. Adult lactating women were invited to participate in the first health check of the newborn (days postpartum 2–5) at the ´Mother and Healthy Children Outpatient Service´, Instituto de Desarrollo e Investigaciones Pediátricas—IDIP, HIAEP “Sor María Ludovica”, La Plata, Argentina between May 2019 and February 2020. Only mothers who were exclusively breastfeeding their infants and expressed the intention to continue breastfeeding exclusively for at least 6 months were included. Exclusion criteria were diabetes (Type I or II or gestational), multiple pregnancy, hypertensive disorders during pregnancy, or delivery of pre-term infant (<37 weeks). Women who agreed to participate arranged a first study visit to the outpatient service at day 10 postpartum (range: days postpartum 5–14) and a second study visit at 3 months postpartum, after an 8 h fasting. The first study visit included blood and milk sampling, anthropometric measurements of both mother and infant and data collection from the maternal clinical record including parity, weight in the first trimester, and weight in the week previous to delivery. First-trimester weight was used to determine the pre-pregnancy body mass index (BMI). Gestational weight gain (GWG) was calculated as the difference between maternal weight at delivery and maternal weight at the first trimester and was dichotomized as *adequate* if weight gain was below or within the Institute of Medicine (IOM) guidelines [26], and *non-adequate* if it exceeded the IOM guidelines. Postpartum weight retention was calculated as maternal weight measured at the 3-month postpartum visits minus pre-pregnancy weight. The second study visit included blood and milk sampling, mother and infant anthropometric measurements, and infant saliva sampling. Infants not exclusively breastfed at 3 months were excluded in the subsequent analysis.

### Sample collection and analysis

Blood, milk, and saliva sampling was standardized between 8 and 10 a.m., as this corresponds to the highest plasma [7] and milk [4] cortisol concentration of the day. For milk sampling, women provided a single, complete breast expression 3 h after the last breastfeeding using a sterile collection shield and container attached to an electric breast pump (NUK Luna Electric Breast Pump, Germany), as described [20]. Skim milk was generated by spinning milk at 3000 × *g* for 20 min [20]. A blood sample was taken from the mother, and plasma was immediately separated. A sample of infant saliva was collected directly from the mouth using a Pasteur pipette. Saliva secretion was stimulated by gentle massage of the salivary glands or with 5% citric acid. The sample was centrifuged at 8000 × *g* for 5 min [27]. Cortisol was detected in maternal plasma, skim milk, and infant saliva by chemiluminescence immunoassay (Access 2, Beckman Coulter, intra-assay CV 5.2%–4.2% and inter-assay CV < 10%). Skim milk was used to avoid lipid interference and reduce matrix effect, as it was reported that cortisol concentrations are similar in whole and skim milk [28]. This technique has low cross-reactivity with substances similar in structure to cortisol.

### Anthropometric measurements

Maternal body weight and body composition, including fat percentage, were measured in duplicate on a bioimpedance scale (Omron HBF514, Argentina) at both visits. Maternal height was also measured. BMI was calculated by dividing weight by height squared (kg/m^2^). Infant anthropometric data included body weight and length as well as left upper arm circumference and triceps and subscapular skinfold thicknesses measured at both visits. Skinfold thicknesses were measured by 2 trained researchers in duplicate using a calibrated Lange caliper (Cambridge, USA). Length, weight, BMI values, arm circumference, and skinfold thicknesses were converted to sex- and age-specific standard deviation scores (*z*-scores) using WHO Anthro software version 1.0.4 (World Health Organization). Birthweight for gestational age (BW/GA) *z*-score was calculated using Fenton’s chart [29]. Standard measurement procedures and techniques were applied in anthropometric measurements [30, 31].

### Statistical methods

Sample size was calculated using G*Power 3.1.9.7 software, to ensure adequate power for detecting differences in milk cortisol concentration between paired samples. The calculation assumed a mean concentration of 0.156 ug/dl and a standard deviation of 0.290 ug/dl, based on previous results from van der Voorn et al. [4], with 90% statistical power and a 5% significance level. These calculations led to a minimal sample size of 33 dyads. Statistical analyses were performed using R statistical software version 4.3.0. Shapiro–Wilk test was applied to test for normality. Continuous variables with normal distribution are presented as mean (standard deviation (SD)); non-normal variables are reported as median (Q1, Q3), and categorical data are summarized as frequency and percentages. Variables presenting a log-normal distribution were log-transformed for subsequent analyses and expressed as geometric mean (95% confidence interval (CI)). The difference between cortisol concentration at 10 days and 3 months postpartum was analyzed by *t*-test. Multiple linear regression analysis, classic or robust, was used to test the relationship of cortisol concentrations with maternal and infant characteristics, adjusting for potential confounders (maternal age, parity (primipara vs. multipara), adequacy of GWG, pre-pregnancy maternal BMI or birthweight for gestational age *z*-score), when needed. Regression coefficients were presented with the corresponding 95% CI. Mediation analysis was conducted to determine whether the relationship between maternal plasma cortisol and infant anthropometric measurements or salivary cortisol concentration was mediated by milk cortisol. To qualify as a mediator, the following criteria needed to be met: [1] the exposure variable (maternal plasma cortisol) must be significantly related to the outcome variable (infant BMI *z*-score or salivary cortisol concentration) (total effect = Path *c*); [2] the exposure variable must show a significant association with the mediator (milk cortisol) (Path *a*); [3] the mediator must be significantly related to the outcome variable (Path *b*); and [4] after adjusting for the mediator, the previously significant relationship between the exposure and outcome variables must no longer be significant (Direct Effect *c*’) [32]. The direct effect was defined as the impact of the exposure variable on the outcome variable without accounting for the influence of the mediator. The indirect or mediation effect was defined as the product of Path *a* and Path *b*, and the total effect equals the sum of the direct and indirect effects (Supplemental Fig. 1) [24]. Linear regression analyses were conducted to examine these relationships, and the mediation effect was tested for significance using the nonparametric Bootstrap CI with the Percentile Method, with a 95% CI [25, 33], performed using the *Mediation* package [34]. All statistical tests were two-tailed and the significance level was set at *p* < 0.05.

## Results

### Cohort characteristics

Sixty-eight mother/infant dyads were enrolled in the study at day 10 postpartum. Mothers had a mean age (range) of 26 [18–37] years and a mean BMI (range) of 27.73 (20.79–43.40) kg/m^2^. Infants had a mean (range) birthweight of 3432 (2380–4350) g, and 52.47% were females. Thirty-four dyads were followed up to 3 months, when mothers had a mean BMI (range) of 26.18 (19.68–44.92) kg/m^2^ and infants had a mean (range) body weight of 6305 (4920–8150) g. Cohort characteristics are presented in Table 1.

**Table 1:** Descriptive characteristics of the study cohort.

	Mean/Median/*n*	Standard deviation/Q1–Q2/%
Maternal characteristics	*n* = 68	
Pre-pregnancy BMI (kg/m^2^)^1^	25.01	22.65, 28.68
Parity, primipara^2^	20	29.41%
IOM GWG guidelines^2^		
Below or within	41	60.29%
Exceed	27	39.71%
At the moment of the visit		
Age (years)^3^	26.07	5.15
BMI (kg/m2)^3^	27.73	4.81
Total adiposity (%)^2^	32.30	4.29
At 3 months postpartum	*n* = 34	
BMI (kg/m2)^1^	26.18	23.20, 29.13
Total adiposity (%)^1^	31.06	27.12, 34.55
Infant characteristics		
At birth	*n* = 68	
Gestational age (weeks)^1^	39	38, 40
Weight (g)^3^	3432.27	454.48
BW/GA *z*-score^3^	0.27	0.80
Length (cm)^1^	50.00	48.00, 51.00
Sex (females)^2^	35	52.47%
At 10 days old	*n* = 68	
Weight (g)^2^	3427.65	452.35
Length (cm)^1^	50.00	48.70, 51.05
BMI *z*-score^2^	0.15	0.91
Length *z*-score^2^	−0.82	1.04
Weight *z*-score^2^	−0.34	0.97
Triceps + Subscapular skinfold (mm)^1^	10.00	8.00, 11.50
At 3 months old	*n* = 34	
Weight (g)^2^	6305.62	710.73
Length (cm)^2^	60.46	2.00
BMI *z*-score^2^	0.32	0.98
Length *z*-score^2^	−0.27	0.91
Weight *z*-score^2^	0.08	0.91
Triceps skinfold *z*-score^2^	−0.12	0.93
Subscapular skinfold *z*-score^2^	0.84	0.95
Triceps + Subscapular skinfold (mm)^2^	18.75	2.89
Salivary Cortisol (ug/dl)^1^	1.32	1.03, 1.98

Data presented as median (Q1, Q3) (1), frequency (%) (2), or mean (SD) (3).

*BMI* body mass index, *IOM GWG guidelines* Institute of Medicine guidelines for gestational weight gain adequacy, *BW/GA z-score* birthweight for gestational age *z*-score.

### Maternal plasma and milk cortisol concentrations are inversely associated with GWG and postpartum weight retention

At 10 days postpartum, plasma cortisol concentration was negatively related to BMI, but this association did not remain significant after adjustments (Table 2). Both plasma and milk cortisol concentrations were inversely associated with GWG and postpartum weight retention at 10 days, but these associations were not observed at 3 months postpartum (Table 2). Additionally, cortisol concentrations were not associated with pre-pregnancy BMI or body fat at either time point (Table 2). Detailed results from unadjusted and adjusted multiple linear regression models are presented (Table 2).

**Table 2:** Associations between maternal cortisol concentrations with pre-pregnancy BMI and percentage de body fat at 10 days and 3 months postpartum.

	Unadjusted analysis		Adjusted analysis^a^	
	Beta (95% CI)	*p* value	Beta (95% CI)	*p* value
10 days postpartum				
Plasma cortisol (ug/dl)				
Pre-pregnancy BMI (kg/m^2^)	0.00 (−0.02, 0.02)	0.734	−0.01 (−0.03, 0.01)	0.455
GWG (kg)	−0.02 (−0.04, −0.01)	**0.009**	−0.02 (−0.03, 0.00)	**0.019**
Postpartum weight retention (kg)	−0.02 (−0.04, −0.01)	**0.003**	−0.02 (−0.04, 0.00)	**0.013**
BMI (kg/m^2^)	−0.02 (−0.04, 0.00)	**0.047**	−0.01 (−0.03, 0.01)	0.176
Body fat (%)	−0.02 (−0.04, 0.00)	**0.039**	−0.01 (−0.03, 0.01)	0.232
Milk cortisol (ug/dl)				
Pre-pregnancy BMI (kg/m^2^)^b^	−0.00 (−0.04, 0.03)	0.791	0.00 (−0.03, 0.03)	0.898
GWG (kg)^b^	−0.02 (−0.04, −0.01)	**0.008**	−0.02 (−0.04, 0.00)	**0.018**
Postpartum weight retention (kg)^b^	−0.03 (−0.05, −0.01)	**<0.001**	−0.03 (−0.05, −0.01)	**0.002**
BMI (kg/m^2^)^b^	−0.02 (−0.05, 0.01)	0.122	−0.02 (−0.04, 0.00)	0.129
Body fat (%)^b^	0.00 (−0.03, 0.02)	0.829	0.00 (−0.02, 0.03)	0.806
3 months postpartum				
Plasma cortisol (ug/dl)				
Pre-pregnancy BMI (kg/m^2^)	−0.30 (−0.62, 0.03)	0.074	−0.23 (−0.59, 0.13)	0.203
GWG (kg)	−0.20 (−0.53, 0.13)	0.227	−0.13 (−0.46, 0.19)	0.408
Postpartum weight retention (kg)	−0.02 (−0.29, 0.26)	0.891	0.11 (−0.19, 0.40)	0.452
BMI (kg/m^2^)	−0.13 (−0.41, 0.15)	0.360	−0.02 (−0.31, 0.27)	0.890
Body fat (%)	−0.05 (−0.26, 0.15)	0.594	−0.04 (−0.28, 0.20)	0.738
Milk cortisol (ug/dl)				
Pre-pregnancy BMI (kg/m^2^)	−0.01 (−0.05, 0.02)	0.409	−0.01 (−0.05, 0.03)	0.579
GWG (kg)	0.00 (−0.03, 0.03)	0.964	0.01 (−0.02, 0.04)	0.631
Postpartum weight retention (kg)	0.01 (−0.02, 0.03)	0.530	0.02 (−0.01, 0.04)	0.287
BMI (kg/m^2^)	0.00 (−0.03, 0.03)	0.990	0.02 (−0.01, 0.05)	0.154
Body fat (%)	0.00 (−0.02, 0.02)	0.982	0.01 (−0.01, 0.03)	0.415

Data are presented as the regression coefficient beta (95% CI) from linear regression analyses. Values in bold indicate statistical significance.

^a^Adjusted for maternal age and parity (primipara vs. multipara).

^b^Robust linear regression analysis.

### Maternal plasma cortisol concentration decreases from 10 days to 3 months postpartum and is associated with milk cortisol concentration

Maternal plasma cortisol concentration was significantly lower at 3 months compared to 10 days postpartum (Fig. 1B), while milk cortisol concentration remained unchanged (Fig. 1C), as determined by Student’s *t* test. Milk cortisol was positively related to plasma concentration at 10 days (Beta (95% CI): 0.75 (0.39, 1.12), *p* < 0.001) and 3 months postpartum (Beta (95% CI): 0.08 (0.05, 0.10), *p* < 0.001) after adjusting for maternal age, pre-pregnancy BMI, parity (primipara vs. multipara) and adequacy of GWG, as determined by multiple linear regression analysis.

### Maternal plasma and milk cortisol are inversely associated with infant anthropometric characteristics

**Fig. 2:**
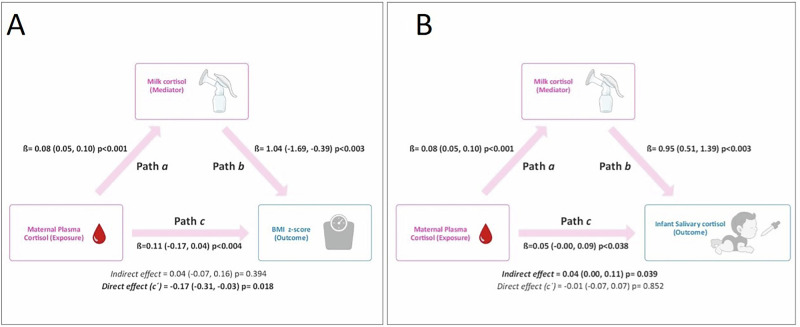
Mediation analyses. Mediation models of the pathway from maternal cortisol plasma concentration to infant BMI *z*-score (**A**) and salivary cortisol concentration (**B**) via milk cortisol. Path *a* indicates the path from maternal plasma cortisol (exposure) to milk cortisol concentrations (mediator). Path *b* indicates the path from milk cortisol concentration (mediator) to infant BMI *z*-score (outcome). Path *c* (Total effect) indicates the path from maternal plasma cortisol concentration (exposure) to infant BMI *z*-score (**A**) or salivary cortisol concentration (**B**) (outcomes). The indirect effect can be estimated by multiplying Path *a* and Path *b*. Direct effect (*c*′) indicates the path from maternal plasma cortisol (exposure) to infant BMI *z*-score (outcome) when controlled for milk cortisol concentration (mediator), this can be estimated as [Path c – (Path a*Path b)]. The models were adjusted for birthweight for gestational age *z*-score, maternal age, pre-pregnancy maternal body mass index, parity (primipara vs. multipara) and adequacy of gestational weight gain. Values in bold indicate statistical significance.

At 10 days postpartum, maternal body fat was inversely associated with infant weight and BMI *z*-scores; plasma cortisol was inversely related to infant arm circumference, but this association did not remain significant after adjustments in multiple linear regression models (Table 3). Milk cortisol was inversely related to arm circumference after adjustments (Table 3). GWG and postpartum weight retention were not associated with infant anthropometrics (data not shown). At 3 months postpartum, maternal BMI and body fat were negatively associated with arm circumference and plasma cortisol was inversely related to arm circumference and triceps skinfold *z*-score after adjustments (Table 3). Milk and plasma cortisol were inversely associated with infant BMI *z*-score after adjustments, therefore a mediation analysis was conducted to determine whether the association between maternal plasma cortisol concentration and infant BMI *z*-score was mediated by milk cortisol (Fig. 2A). When milk cortisol was added to the model as a control variable, the association between maternal plasma and infant salivary concentrations remained significant (Direct Effect *c*’, *p* = 0.018). The analysis showed that milk cortisol did not significantly mediate the association between maternal plasma cortisol concentration and infant BMI *z*-score (indirect effect *p* = 0.394). Despite maternal BMI and body fat being inversely associated with infant anthropometric characteristics, mediation analysis was not conducted due to a lack of association of the former with milk cortisol concentration (Table 2).

**Table 3:** Associations of maternal anthropometric characteristics and cortisol concentrations with infant anthropometric measurements at day 10 postpartum and at 3 months old.

	Unadjusted analysis		Adjusted analysis^b^		Adjusted analysis^c^	
	Beta (95% CI)	*p* value	Beta (95% CI)	*p* value	Beta (95% CI)	*p* value
10 days postpartum						
Maternal BMI (kg/m^2^)						
Weight *z*-score	0.04 (−0.01, 0.08)	0.133	−0.01 (−0.16, 0.13)	0.846	−0.05 (−0.14, 0.04)	0.243
BMI *z*-score	−0.01 (−0.06, 0.04)	0.728	−0.08 (−0.22, 0.06)	0.280	−0.10 (−0.20, 0.01)	0.079
Arm circumference (mm)^a^	0.04 (0.00, 0.08)	0.069	0.05 (−0.08, 0.19)	0.413	−0.05 (−0.11, 0.10)	0.744
Triceps + Subscapular skinfold (mm)^a^	0.01 (−0.08, 0.10)	0.814	−0.13 (−0.43, 0.16)	0.370	−0.18 (−0.46, 0.09)	0.182
Maternal body fat (%)						
Weight *z*-score	0.02 (−0.02, 0.07)	0.312	−0.04 (−0.13, 0.05)	0.372	−0.07 (−0.12, −0.01)	**0.019**
BMI *z*-score	0.00 (−0.04, 0.04)	0.966	−0.05 (−0.13, 0.02)	0.170	−0.06 (−0.12, −0.01)	**0.027**
Arm circumference (mm)^a^	0.04 (0.00, 0.08)	**0.035**	0.00 (−0.07, 0.07)	0.965	0.01 (−0.05, 0.06)	0.838
Triceps + Subscapular skinfold (mm)^a^	0.01 (−0.07, 0.09)	0.744	−0.02 (−0.18, 0.13)	0.777	−0.05 (−0.19, 0.09)	0.460
Maternal plasma cortisol (ug/dl) (log)						
Weight *z*-score	−0.48 (−1.01, 0.05)	0.072	−0.39 (−0.94, 0.19)	0.186	−0.22 (−0.58, 0.13)	0.215
BMI *z*-score^d^	−0.41 (−0.96, 0.13)	0.144	−0.41 (−1.25, 0.43)	0.340	−0.32 (−1.22, 0.57)	0.479
Arm circumference (mm)^a^	**−0.66 (−1,15, −0.19)**	**0.007**	−0.45 (−0.94, 0.05)	0.075	−0.29 (−0.68, 0.10)	0.140
Triceps + Subscapular skinfold (mm)^a,d^	0.15 (−0.91, 1.21)	0.777	0.37 (−1.42, 2.16)	0.686	0.63 (−1.07, 2.33)	0.469
Milk cortisol (ug/dl) (log)						
Weight *z*-score	−0.17 (−0.53, 0.19)	0.348	−0.11 (−0.47, 0.25)	0.550	−0.07 (−0.29, 0.16)	0.553
BMI *z*-score	−0.28 (−0.63, 0.07)	0.116	−0.28 (−0.65, 0.08)	0.125	−0.18 (−0.47, 0.10)	0.196
Arm circumference (mm)^a^	−0.36 (−0.90, 0.18)	0.194	−0.26 (−0.65, 0.13)	0.191	**−0.36 (−0.68, −0.05)**	**0.029**
Triceps + Subscapular skinfold (mm)^a^	0.22 (−0.93, 1.37)	0.704	0.18 (−1.42, 1.78)	0.823	0.28 (−0.66, 1.23)	0.558
3 months postpartum						
Maternal BMI (kg/m^2^)						
Weight *z*-score	0.06 (−0.02, 0.14)	0.111	0.06 (−0.14, 0.25)	0.556	0.02 (−0.15, 0.18)	0.854
BMI *z*-score	0.03 (−0.04, 0.10)	0.441	0.05 (−0.15, 0.24)	0.638	0.03 (−0.17, 0.22)	0.786
Arm circumference (mm)^a^	0.03 (−0.05, 0.11)	0.429	0.08 (−0.14, 0.30)	0.479	−0.09 (−0.17, −0.01)	**0.036**
Arm circumference *z*-score	−0.01 (−0.06, 0.04)	0.696	−0.09 (−0.26, 0.06)	0.242	−0.14 (−0.28, 0.00)	**0.043**
Triceps + Subscapular skinfold (mm)^a,d^	−0.03 (−0.20, 0.14)	0.715	0.19 (−0.48, 0.85)	0.572	0.18 (−0.41, 0.77)	0.532
Triceps skinfold *z*-score	0.04 (−0.06, 0.13)	0.449	0.05 (−0.15, 0.25)	0.604	0.01 (−0.18, 0.20)	0.929
Subscapular skinfold *z*-score	0.06 (−0.04, 0.16)	0.205	0.19 (−0.01, 0.40)	0.068	0.19 (−0.02, 0.41)	0.080
Maternal body fat (%)						
Weight *z*-score^d^	0.04 (−0.01, 0.08)	0.099	0.04 (−0.04, 0.12)	0.331	0.02 (−0.05, 0.09)	0.609
BMI *z*-score	0.04 (−0.01, 0.09)	0.117	0.05 (−0.04, 0.14)	0.246	0.04 (−0.05, 0.13)	0.359
Arm circumference (mm)^a^	−0.02 (−0.06, 0.03)	0.453	−0.04 (−0.13, 0.05)	0.383	−0.08 (−0.16, 0.00)	**0.039**
Arm circumference *z*-score	−0.01 (−0.05, 0.02)	0.456	−0.04 (−0.12, 0.03)	0.241	−0.07 (−0.13, −0.01)	**0.031**
Triceps + Subscapular skinfold (mm)^a^	0.05 (−0.10, 0.20)	0.480	0.41 (0.12, 0.69)	**0.007**	0.39 (0.09, 0.68)	**0.013**
Triceps skinfold *z*-score	0.00 (−0.05, 0.05)	0.992	0.09 (0.01, 0.18)	**0.031**	0.07 (−0.01, 0.15)	0.101
Subscapular skinfold *z*-score^d^	0.03 (−0.01, 0.07)	0.145	0.10 (0.01, 0.20)	**0.031**	0.11 (0.01, 0.20)	**0.029**
Maternal plasma cortisol (ug/dl)						
Weight *z*-score^d^	−0.07 (−0.15, 0.01)	0.087	−0.07 (−0.29, 0.16)	0.571	−0.09 (−0.19, 0.01)	0.075
BMI *z*-score	**−0.10** (**−0.18, −0.02)**	**0.019**	**−0.11** (**−0.18, −0.03)**	**0.006**	**−0.11** (**−0.17, −0.04)**	**0.004**
Arm circumference (mm)^a^	−0.06 (−0.15, 0.02)	0.144	−0.08 (−0.17, 0.01)	0.076	**−0.09 (−0.17, −0.01)**	**0.036**
Arm circumference *z*-score	−0.06 (−0.14, 0.01)	0.093	−0.07 (−0.15, 0.01)	0.097	**−0.09 (−0.17, −0.02)**	**0.017**
Triceps + Subscapular skinfold (mm)^a^	−0.03 (−0.55, 0.48)	0.895	−0.10 (−0.64, 0.44)	0.703	−0.15 (−0.70, 0.40)	0.574
Triceps skinfold *z*-score	−0.04 (−0.12, 0.03)	0.268	−0.08 (−0.16, 0.00)	0.063	**−0.09 (−0.17, −0.01)**	**0.022**
Subscapular skinfold *z*-score	−0.03 (−0.11, 0.05)	0.883	0.00 (−0.08, 0.08)	0.957	0.00 (−0.08, 0.08)	0.909
Milk cortisol (ug/dl) (log)						
Weight *z*-score	−0.35 (−1.12, 0.42)	0.357	0.10 (−0.82, 1.02)	0.819	−0.23 (−1.05, 0.59)	0.572
BMI *z*-score	−0.50 (−1.12, 0.11)	0.102	**−0.94 (−1.60, −0.27)**	**0.008**	**−1.04 (−1.69, −0.39)**	**0.003**
Arm circumference (mm)^a^	−0.26 (−1.08, 0.56)	0.516	−0.25 (−1.19, 0.69)	0.584	−0.38 (−1.31, 0.55)	0.410
Arm circumference *z*-score	0.06 (−0.74, 0.86)	0.885	0.04 (−0.86, 0.93)	0.935	−0.09 (−0.92, 0.74)	0.831
Triceps + Subscapular skinfold (mm)^a^	−0.60 (−3.06, 1.86)	0.634	−0.76 (−3.30, 1.79)	0.566	−0.98 (−4.78, 1.60)	0.618
Triceps skinfold *z*-score	0.08 (−0.79, 0.95)	0.852	0.03 (−0.91, 0.97)	0.948	−0.09 (−0.95, 0.77)	0.830
Subscapular skinfold *z*-score	−0.25 (−1.01, 0.51)	0.501	−0.28 (−1.09, 0.53)	0.485	0.04 (−0.91, 1.00)	0.932

Data are presented as the regression coefficient beta (95% confidence interval) from linear regression analyses. Values in bold indicate statistical significance.

^a^Arm circumference and sum of skinfolds adjusted for infant age and sex.

^b^Adjusted for maternal age, pre-pregnancy maternal body mass index, parity (primipara vs. multipara) and adequacy of gestational weight gain.

^c^Adjusted for birthweight for gestational age *z*-score, maternal age, pre-pregnancy maternal body mass index, parity (primipara vs. multipara) and adequacy of gestational weight gain.

^d^Robust linear regression analysis.

### Infant salivary cortisol is associated with maternal cortisol concentration but not with anthropometric characteristics

Infant saliva cortisol was positively related to maternal plasma and milk cortisol but not with infant BMI *z*-score, arm circumference, triceps, and subscapular skinfold *z*-score at 3 months old (Table 4). A mediation analysis was conducted to determine whether the association between maternal plasma cortisol and infant salivary concentration was mediated by milk cortisol (Fig. 2B), revealing an indirect effect (*p* = 0.039). When milk cortisol was added to the model as a control variable, the association between maternal plasma and infant salivary cortisol concentrations was no longer significant (direct effect *c*’, *p* = 0.852).

**Table 4:** Associations between infant salivary cortisol concentrations with maternal cortisol concentrations and anthropometric measurements at 3 months old.

	Unadjusted analysis		Adjusted analysis^b^		Adjusted analysis^c^	
	Beta (95% CI)	*p* value	Beta (95% CI)	*p* value	Beta (95% CI)	*p* value
Infant salivary cortisol (µU/dl, log)						
Maternal cortisol concentrations						
Plasma cortisol (µU/dl)	0.05 (0.00, 0.09)	**0.031**	0.05 (0.00, 0.09)	**0.034**	0.05 (0.00, 0.09)	**0.038**
Milk cortisol (µU/dl, log)	0.96 (0.57, 1.35)	**0.001**	0.96 (0.53, 1.38)	**<0.001**	0.95 (0.51, 1.39)	**<0.001**
Infant anthropometric measurements						
Weight *z*-score	0.10 (−0.39, 0.58)	0.683	0.11 (−0.38, 0.60)	0.648	0.04 (−0.41, 0.49)	0.861
BMI *z*-score	−0.25 (−0.75, 0.25)	0.315	−0.29 (−0.77, 0.19)	0.233	−0.32 (−0.80, 0.15)	0.173
Arm circumference (mm)^a^	−0.03 (−0.62, 0.56)	0.907	−0.02 (−0.66, 0.62)	0.950	−0.04 (−0.67, 0.59)	0.903
Arm circumference *z*-score	0.10 (−0.37, 0.58)	0.657	0.12 (−0.40, 0.64)	0.641	0.10 (−0.37, 0.57)	0.667
Triceps + Subscapular skinfold (mm)^a^	−0.52 (−2.48, 1.43)	0.589	−0.44 (−2.57, 1.69)	0.674	−0.25 (−2.38, 1.87)	0.805
Triceps skinfold *z*-score	−0.42 (−1.04, 0.20)	0.173	−0.41 (−1.06, 0.23)	0.203	−0.38 (−1.00, 0.24)	0.223
Subscapular skinfold *z*-score	0.24 (−0.34, 0.81)	0.408	0.22 (−0.39, 0.84)	0.466	0.26 (−0.33, 0.85)	0.374

Data are presented as the regression coefficient beta (95% confidence interval) from linear regression analyses. Values in bold indicate statistical significance.

^a^Arm circumference and sum of skinfolds adjusted for infant age and sex.

^b^Adjusted for maternal age, pre-pregnancy maternal body mass index, parity (primipara vs. multipara) and adequacy of gestational weight gain.

^c^Adjusted for birthweight for gestational age *z*-score, maternal age, pre-pregnancy maternal body mass index, parity (primipara vs. multipara) and adequacy of gestational weight gain.

## Discussion

To our knowledge, this is the first study to examine the effects of maternal plasma and milk cortisol, as well as infant salivary cortisol, on infant body weight and adiposity within a single cohort. Our results show that maternal plasma and milk cortisol are inversely associated with infant BMI *z*-score at 3 months of lactation. Additionally, our findings indicate that maternal cortisol levels are associated with infant salivary concentration, with this relationship mediated by milk cortisol. These results uncover novel roles for cortisol in the context of human lactation.

The concentration of milk bioactive components, including cortisol, depends on diverse maternal characteristics [14, 18–20, 35]. Maternal weight status is a factor that can affect the concentration of human milk hormones. Our findings show a transient negative association of milk cortisol with specific indicators of maternal weight status such as GWG and postpartum weight retention, observed in the early postpartum but not at 3 months, but no relationship with pre-pregnancy BMI, postpartum BMI, or adiposity. Our results confirm previous findings about the lack of influence of pre-pregnancy BMI on milk cortisol in different stages of lactation [9, 36, 37]. Still, data about the association of milk cortisol with current weight status during lactation is scarce and contradictory [9, 10]. One study reports a direct association of milk cortisol with maternal BMI but not with adiposity [10], while others report no association [9, 36]. Similarly, the association of plasma cortisol with anthropometric measures in subjects with obesity yields inconclusive results. A systematic review of studies examining cortisol activity in obesity [38] indicated that higher abdominal fat is generally associated with increased cortisol reactivity; however, some studies within the review reported blunted cortisol responses. Our study did not reveal any association between waist-to-hip ratio and milk or plasma cortisol (data not shown). These findings suggest that the relationship between circulating cortisol and body fat-related anthropometric measures remains unclear and warrants further investigation, particularly in lactating women.

It is well established that the levels of bioactive components in milk, including cortisol, can fluctuate across different stages of lactation [39, 40]. Here we observed that maternal plasma cortisol levels declined by 3 months postpartum, aligning with previous reports documenting a decrease from late pregnancy to the third month of lactation [41, 42]. Interestingly, in contrast to the changes observed in plasma, milk cortisol concentrations remained stable during this period, confirming earlier findings [9, 10, 43]. These results indicate that, despite the reduction in circulating cortisol, its concentration in milk does not vary. Furthermore, we found a positive correlation between milk and plasma cortisol concentrations, with a consistent milk/plasma ratio of approximately 0.10, in agreement with previous observations in the first month postpartum [4, 44]. Our findings align with the idea that, due to its lipophilic structure, cortisol enters the milk via passive diffusion through the mammary epithelium, following its concentration gradient [35, 44, 45]. These observations highlight the tight regulation of cortisol transfer into breast milk, ensuring stable exposure to the infant despite fluctuations in maternal circulating levels.

Little is known about whether milk hormones can regulate the infant hormonal milieu. Milk leptin has been shown to correlate with leptin concentrations in infant circulation [46], however, it is unknown whether maternal cortisol may regulate endogenous cortisol levels in the infant. Our findings are the first suggesting that maternal plasma and milk cortisol influence infant salivary concentration, and this effect is mediated through milk cortisol. Our results are in line with reports of a direct relationship between cortisol in maternal circulation and cord blood [47], and between maternal and infant salivary cortisol concentrations [48, 49], although other authors report no association between the mother-child dyad [50]. Several potential mechanisms may be involved in the mediation effect of milk cortisol on infant concentration: once absorbed, milk cortisol could enter infant circulation and influence the HPA axis activity via negative feedback regulation [51], or lead to epigenetic modifications in stress-regulation genes, affecting long-term HPA axis function [52]. Our findings highlight the role of maternal cortisol in the regulation of infant levels.

Given the role of cortisol in controlling metabolism, exposure via human milk may contribute to the early metabolic programming of body weight [11]. Here, we identified that high maternal cortisol concentrations in milk and plasma were associated with lower infant BMI *z*-score at 3 months. In line with our findings, a recent Mendelian Randomization study shows that higher maternal morning plasma cortisol causes lower birth weight [53]. The fact that the association between maternal plasma cortisol and infant BMI *z*-score is not mediated by milk cortisol content suggests that elevated maternal cortisol levels may exert a direct negative effect on infant body weight, independent of milk cortisol. The postpartum period is characterized by significant physiological and psychological changes in mothers. Numerous prenatal and postnatal factors can acutely influence maternal stress levels, leading to elevated circulating cortisol concentrations[54]. These increased cortisol levels can impact not only infant behavior [6, 22] but also body weight, as demonstrated by our results. Few studies have addressed the role of milk cortisol on infant body weight and adiposity, with inconclusive results. Two studies report a lack of association between milk cortisol and infant growth or body composition at 3 months of lactation [8, 9], and one study shows a positive association with infant fat mass in the first year of life [10]. In line with our results, Hahn-Holbrook et al. [11] found a negative association between milk cortisol in the third month of lactation on BMI percentile gain in the first 2 years of life. Possible discrepancies between studies may result from differences in sample collection (the number of samples and timing of collection), sample size, breastfeeding (exclusive vs. partial), and cortisol analysis methods. Another possible explanation for the different findings is that some studies did not fully adjust for potential confounders of the tested associations. Moreover, animal and in vitro studies show that glucocorticoid ingestion facilitates intestinal maturation [55, 56], and although this effect has yet to be determined in infants, it could potentially be involved in the beneficial effects of breastfeeding. Our results also show that the associations between maternal cortisol and infant BMI *z*-score and adiposity were observed at 3 months but not in the early stages of lactation, suggesting that the effect of maternal cortisol on infant body weight and fat accretion could vary over time. Therefore, our results and others indicate a negative impact of maternal cortisol on infant body weight and adiposity at 3 months of lactation.

The first months of life are considered a critical window for growth and adiposity programming [12, 13]. Leptin and ghrelin plasma concentrations have been reported to be positively associated with infant fat mass during the first 6 months of life [12, 15], while plasma LEAP2 was directly related to BMI *z*-score [14] in exclusively breastfed infants. Our results show that infant salivary cortisol, which has been reported to be directly related to its plasma concentration [57], is not associated with body weight and adiposity at 3 months of lactation. These results are in line with reports showing that cortisol concentrations in infant saliva upon waking were not associated with BMI in infants aged 2 to 12 months [58], and with a recent systematic review indicating that morning salivary cortisol concentration is not associated with growth and adiposity in children 4 to 12 years old [16]. This observation may indicate that infant salivary cortisol is not involved in body weight and fat accretion during lactation.

The main strengths of our study include the assessment of maternal plasma and milk cortisol and also infant salivary concentration within a single cohort of exclusively breastfed infants. Although we did not collect milk samples throughout the day, sample collection was standardized to capture the peak cortisol concentration. Also, our results show models adjusted by maternal and infant confounders. These rigorous study conditions allowed us to conduct a comprehensive analysis of the potential role of maternal cortisol in a single study. It is important to contextualize the limitations of this study, given the complexity of follow-up in longitudinal clinical studies. The sample size decreased by the second visit, as several mother-child dyads were lost mainly due to the introduction of formula feeding or the COVID-19 pandemic. Therefore, the small sample size may lead to insufficient power for detecting certain associations between cortisol concentration and anthropometric variables. Also, an immunometric assay was used to assess cortisol concentrations, which does not permit simultaneous determination of cortisone. Likewise, collecting only one sample per visit does not allow us to observe the diurnal pattern of cortisol concentration in milk and circulation [59]. Despite these limitations, our results are valuable to shed light on the role of milk cortisol in infant growth.

In conclusion, our study shows that high maternal cortisol concentrations are inversely associated with infant BMI *z*-score during lactation. Situations that affect maternal cortisol concentrations, like postpartum stress, may negatively affect infant BMI *z*-score. Further research is warranted to explore the mechanisms through which milk cortisol exerts its effects on infants and how these interactions evolve across different stages of lactation.

## Supplementary information

supplemental material

## Data Availability

Data described in the manuscript, code book, and analytic code will be made available upon request.
