# Supplementary material for: Human milk cortisol is inversely associated with infant BMI and mediates the association between maternal plasma and infant salivary cortisol concentrations
Source: Int J Obes (Lond). 2025 May 31;49(8):1632–41. doi: 10.1038/s41366-025-01815-4 (PMC12396966; doi:10.1038/s41366-025-01815-4)
Supplement: Supplementary file 1 — supplemental material [file 41366_2025_1815_MOESM1_ESM.docx]

**Human milk cortisol is inversely associated with infant BMI and mediates the association between maternal plasma and infant salivary cortisol concentrations.**

Ana Luz Kruger^1,2^, Agustina Malpeli^1^, Marisa Sala^1^, Carla Casado^1^, Ignacio Mendez^1^, Lucrecia Fotia^1^, Andrea Tournier^3^, María Victoria Fasano^1,4^, María F. Andreoli^1,2,5^

**Supplemental Figure 1:** Conceptual diagram of the investigated mediation analyses.


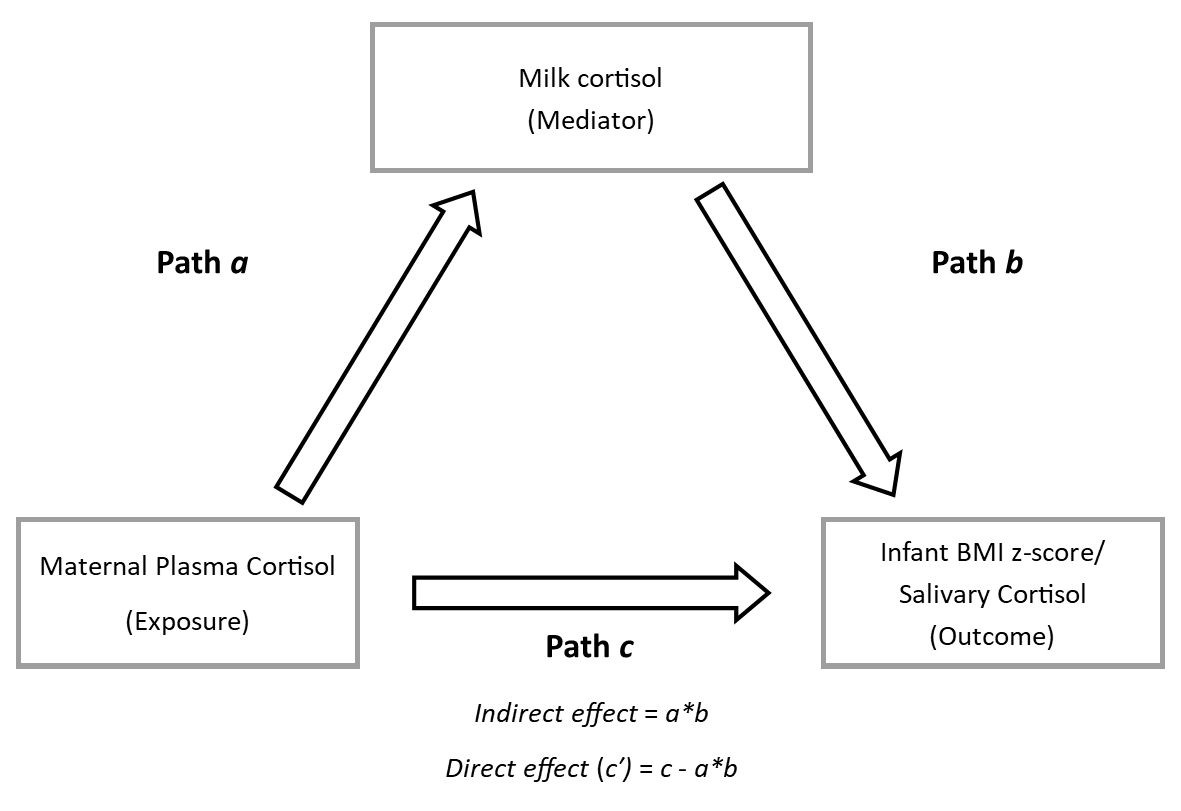


Path *a* indicates the path from maternal plasma cortisol (exposure) to milk cortisol concentrations (mediator). Path *b* indicates the path from milk cortisol concentration (mediator) to infant BMI z-score (outcome). Path *c* (Total effect) indicates the path from maternal plasma cortisol concentration (exposure) to infant BMI z-score (outcome). The indirect effect can be estimated by multiplying Path *a* and Path *b*. Direct effect (*c*′) indicates the path from maternal plasma cortisol (exposure) to infant BMI z-score (outcome) when controlled for milk cortisol concentration (mediator), this can be estimated as [Path c – (Path a*Path b)]. The model was adjusted for birthweight for gestational age z-score, maternal age, pre-pregnancy maternal body mass index, parity (primipara vs. multipara) and adequacy of gestational weight gain.
